# Human amnion epithelial cells rescue cell death via immunomodulation of microglia in a mouse model of perinatal brain injury

**DOI:** 10.1186/s13287-017-0496-3

**Published:** 2017-02-28

**Authors:** Bryan Leaw, Dandan Zhu, Jean Tan, Ruth Muljadi, Mohamed I. Saad, Joanne C. Mockler, Euan M. Wallace, Rebecca Lim, Mary Tolcos

**Affiliations:** 1 The Ritchie Centre, Hudson Institute of Medical Research, 27-31 Wright Street, Clayton, VIC 3168 Australia; 2 Department of Obstetrics and Gynaecology, Monash University, Clayton, VIC 3168 Australia; 3 School of Health and Biomedical Sciences, RMIT University, Bundoora, VIC 3083 Australia

**Keywords:** Amnion cell, Cell therapy, Perinatal brain injury, Brain development, Inflammation, Hyperoxia, Microglia, Immunotherapy

## Abstract

**Background:**

Human amnion epithelial cells (hAECs) are clonogenic and have been proposed to reduce inflammatory-induced tissue injury. Perturbation of the immune response is implicated in the pathogenesis of perinatal brain injury; modulating this response could thus be a novel therapy for treating or preventing such injury. The immunomodulatory properties of hAECs have been shown in other animal models, but a detailed investigation of the effects on brain immune cells following injury has not been undertaken. Here, we investigate the effects of hAECs on microglia, the first immune responders to injury within the brain.

**Methods:**

We generated a mouse model combining neonatal inflammation and perinatal hyperoxia, both of which are risk factors associated with perinatal brain injury. On embryonic day 16 we administered lipopolysaccharide (LPS), or saline (control), intra-amniotically to C57Bl/6 J mouse pups. On postnatal day (P)0, LPS pups were placed in hyperoxia (65% oxygen) and control pups in normoxia for 14 days. Pups were given either hAECs or saline intravenously on P4.

**Results:**

At P14, relative to controls, LPS and hyperoxia pups had reduced body weight, increased density of apoptotic cells (TUNEL) in the cortex, striatum and white matter, astrocytes (GFAP) in the white matter and activated microglia (CD68) in the cortex and striatum, but no change in total microglia density (Iba1). hAEC administration rescued the decreased body weight and reduced apoptosis and astrocyte areal coverage in the white matter, but increased the density of total and activated microglia. We then stimulated primary microglia (CD45^low^CD11b^+^) with LPS for 24 h, followed by co-culture with hAEC conditioned medium for 48 h. hAEC conditioned medium increased microglial phagocytic activity, decreased microglia apoptosis and decreased M1 activation markers (CD86). Stimulating hAECs for 24 h with LPS did not alter release of cytokines known to modulate microglia activity.

**Conclusions:**

These data demonstrate that hAECs can directly immunomodulate brain microglia, probably via release of trophic factors. This observation offers promise that hAECs may afford therapeutic utility in the management of perinatal brain injury.

## Background

Preterm birth is a major, independent, risk factor for neurodevelopmental impairment because of the infant’s immaturity. Most babies born very preterm (<32 weeks) require ventilator support to manage respiratory distress syndrome, and although intervention strategies have greatly improved the survival of these infants, the incidence of bronchopulmonary dysplasia and associated neurodevelopmental problems has not changed dramatically [1, 2]. The pathogenesis of the underlying brain injury is complex, but is thought to involve both inflammation and ischaemia leading to downstream oxidative stress and subsequent neuronal and axonal death [3]. Importantly, the innate immune response plays a critical role in the inflammatory sequelae to hypoxic–ischaemic injury in the brain and therefore in the extent of any subsequent functional loss [4]. The glial response to injury is involved in the pathogenesis of multiple central nervous system (CNS) disorders [5], and microglial activation has been linked to oxidative stress-induced neurotrauma [6]. Microglia are the resident immune cells within the CNS that are the first responders to injury [7]. It is increasingly appreciated that modulation of microglial activation can confer neuroprotection in diverse forms of injury or inflammation [8–10], suggesting that microglia may be a novel therapeutic target for brain injury, including injury during early brain development [11, 12].

Cell therapies have recently attracted much interest as potential treatments for some of the key complications of preterm birth [13–15]. For example, human amnion epithelial cells (hAECs) appear particularly promising for preterm lung injury [13, 16, 17] and amniotic fluid cells for necrotizing enterocolitis [18]. With regard to perinatal brain injury, hAECs have also been shown to reduce lipopolysaccharide (LPS)-induced fetal brain inflammation and subsequent injury [19]. While hAECs express early neural markers [20] and are able to differentiate into neurons, astrocytes and oligodendrocytes in vitro [21], their reparative effects are thought to be primarily exerted via modulation of the host immune response to injury [22]. While no studies to date have examined whether hAECs can modify microglial function in brain injury, the reparative actions of hAECs within the lung are macrophage dependent [23, 24].

There are a myriad of animal models for preterm brain injury, developed both to improve the understanding of the different pathways to injury and to identify new therapeutic targets [25–27]. Rodent models are the most common, due to the extensive amount of literature correlating rodent anatomical and functional development to the appropriate stage in humans [28]. These studies have shown that the stage of brain development of a human infant at birth is roughly equivalent to the neurodevelopment of a rodent at postnatal day (P)12–13 [11]. Thus, studies of pre-weaning rodents (first 2 postnatal weeks) are thought to appropriately mimic stages of human brain development that occur late in utero, or ex utero in the case of severely preterm birth [29]. It is also thought that a two-hit model of brain injury, combining prenatal inflammatory insult and secondary oxidative stress, may best reflect the pathogenesis of common human preterm brain injury [30]. Inflammation and oxidative stress are stressors that together increase the probability of developing white matter injury and associated respiratory disease, both of which are common in very preterm infants [31]. Further, infants exposed to excessively high oxygen saturation levels are at increased risk of complications such as retinopathy of prematurity and bronchopulmonary dysplasia [32]. Despite this, the association of brain injury with combined intrauterine infection and hyperoxia is not well understood. To this end we have used a mouse model of neonatal brain injury that utilizes both intra-amniotic administration of LPS (inflammation) and perinatal chronic hyperoxia. LPS is a widely used insult that induces an innate inflammatory response [33] mimicking fetal or neonatal infection that is a major risk factor for perinatal brain injury [34]. In our model of combined intra-amniotic LPS and perinatal chronic hyperoxia, we aimed to characterize injury within brain regions that are vulnerable to hyperoxia-induced and/or inflammation-induced damage in preterm infants such as the cerebral white matter, cerebral cortex and striatum [6, 35]. We then sought to assess the effect, if any, of hAECs in mitigating these changes with a particular focus on microglial modulation.

## Methods

### Experimental groups

C57Bl6/J mice were allocated randomly into four experimental groups as follows: control animals were given intra-amniotic saline at embryonic day 16 (E16) and born into normoxia (21% O_2_); and hyperoxia groups were given intra-amniotic LPS at E16, and then placed in hyperoxia (65% O_2_) immediately after birth. Mice (both control and LPS + hyperoxia animals) were then administered either saline or hAECs on P4.

### hAEC isolation

Placentae were collected from women with uncomplicated pregnancies undergoing elective caesarean sections at term. Collection and isolation of hAECs was performed as described previously [36]. The mean gestational age of consenting women was 38 weeks. Cells were then cryopreserved using standard methods at 5 × 10^6^ cells/ml. Only isolates with >85% post-thaw cell viability, >90% EpCAM-positive cells and <1% CD90/105-positive cells were used in this study. Freshly thawed hAECs were used for all experiments described in this manuscript (i.e. P1).

### Surgery

Induction of the intra-amniotic inflammation was performed as described previously [25]. Briefly, on E16 pregnant C57Bl/6 J mice were anaesthetized with isofluorane and the amniotic sacs were exteriorized by hysterotomy through a midline abdominal incision. The uterine horn was exposed and LPS from *Escherichia coli* (1 μg in 50 μl saline, 055:B5; Sigma, St. Louis, MO, USA) or 50 μl saline was administered to each amniotic sac. To do so, we used an automated microinjection system (IM 300; Narishige, Tokyo, Japan) with bevelled glass micropipettes pulled using a P-1000 micropipette puller (Sutter Instruments, CA, USA) connected to the microinjection system. The micropipette was physically advanced towards the uterine wall and into the amniotic cavity, and LPS or saline was administered. The micropipettes were then retracted physically, a new amniotic sac positioned and the procedure repeated. After the injections were completed, the uterine horn was placed back into the abdominal cavity, the laparotomy closed and the mother recovered. The overall procedure took less than 30 min per dam, from incision to closure. Pups were then allowed to deliver naturally at term (E21). Control (saline administered) pups were placed into normoxia (21% O_2_) chambers and pups administered intra-amniotic LPS were placed into hyperoxia (65% O_2_) chambers, for 14 days consecutively. We did not experience any mortality in our experimental groups.

### hAEC treatment

At P4 mice were briefly (<60 min) removed from their hyperoxic or normoxic chamber to be administered either normal saline (10 μl) or hAECs (100,000 cells in 10 μl saline) intravenously via the superficial temporal vein, using the automated microinjection system as per the intra-amniotic injections. Pups were then placed back into their respective chambers until P14.

### Perfusion and tissue preparation

At P14 all pups (control, *n* = 9; LPS + hyperoxia, *n* = 7; LPS + hyperoxia + hAECs, *n* = 6) were killed via ketamine–xylazine overdose (200 μl of 100 mg/ml ketamine 10 mg/ml xylazine per 8 g body weight) and transcardially perfused with saline. After clearance of circulating blood, 4% paraformaldehyde (PFA) in 0.1 M phosphate buffer (pH 7.4) was perfused for a further 5 min. Brain tissue was then post-fixed in 4% PFA for 24 h at 4 °C. The cerebral hemispheres were embedded in paraffin and 5-μm-thick serial coronal sections were cut from the anterior pole of the lateral ventricle to the hippocampus. Every eighth section was stained using haematoxylin and eosin for orientation purposes and analysis of gross morphological changes or stained immunohistochemically.

### Immunohistochemistry

Sections from control (*n* = 9), LPS + hyperoxia (*n* = 6) and LPS + hyperoxia + hAEC (*n* = 7) pups were exposed to heat-induced epitope retrieval using citrate buffer (pH 6) and blocked in 2% (w/v) bovine serum albumin in phosphate-buffered saline for 1 h and primary antibody applied overnight at 4 °C in a humid chamber. Immunohistochemistry was performed on paraffin-embedded sections to detect microglia (ionized calcium-binding adaptor molecule 1 (Iba1), 1:1000, 019-19741; Wako Pure Chemical Industries, Osaka, Japan), activated microglia (CD68, 1:100, MCA1957; ABD Serotec, Oxford, UK) and astrocytes (glial fibrillary acidic protein (GFAP), 1:1000, M7240; DAKO, CA, USA), as described previously [37]. hAEC cell tracking was performed using anti-human HLA-G (HLA-G clone 87G, 1:100, ALX-805-719-c100; Enzo Lifesciences, Australia). Biotinylated secondary antibodies (1:200) were applied for 1 h at room temperature in a humid chamber and reacted with the VectaStain Elite avidin–biotin complex kit (1:200; Vector Laboratories, CA, USA) using 3,3′-diaminobenzidine as the chromagen. Following counterstaining with haematoxylin, slides were coverslipped with DPX (Sigma-Aldrich, MO, USA). Omission of the primary antibody resulted in no specific staining. For each antibody, all sections were stained simultaneously to ensure uniform conditions for subsequent analysis.

### TUNEL assay

Sections were labelled using the DeadEnd Colorimetric terminal transferase nick end-labelling (TUNEL) system (Promega, Madison, WI, USA) to identify apoptotic and necrotic cell death [38], as per the manufacturer’s directions. Additional sections were exposed to DNAse I (Roche Molecular Biochemicals) and incubated at 37 °C for 30 min (positive control) or incubated without terminal transferase (negative control).

### Imaging and quantitative analysis

Images were scanned on an Aperio Scanscope XT (Aperio Technologies, CA, USA) for image analysis using ImageScope software (Aperio Technologies) for individual cell counts, and the ImageJ Fiji software package (NIH Image, MD, USA) for calculation of positive staining area. The observer (BL) was blinded to experimental groups to prevent bias. Analysis was done using three replicates per animal and a mean calculated per animal. A mean of means was then determined for each experimental group. For the hAEC tracking experiments, slides were counterstained with DAPI 1:5000 for 15 min, and images were scanned on a Nikon C1 Confocal laser-scanning microscope.

#### Cell density of GFAP-positive, Iba1-positive, CD68-positive and TUNEL-positive cells

GFAP-positive, Iba1-positive, CD68-positive and TUNEL-positive cells were counted in 5-μm-thick coronal sections (separated by 40 μm, three sections per animal). In the striatum (Iba1, CD68), analysis was performed in five random fields of view (objective: 40×; field size 0.04 mm^2^). In the motor-somatosensory cortex (Iba1, CD68), counts were performed in one field divided into three bins corresponding to specific cortical layers (bin 1: layers I/II, bin 2: layers III/IV, bin 3: layer V). In white matter tracts (GFAP, Iba1, CD68), counts were in random fields of view (objective: 40×; field size 0.04 mm^2^) in the corpus callosum (two fields of view), cingulum (two fields of view) and external capsule (three fields of view). TUNEL-positive cells were counted within the entire cross-sectional area of the striatum, cerebral cortex and subcortical white matter. The area of each region or field of view was determined to calculate areal density (cells/mm^2^) of GFAP-positive, Iba1-positive, CD68-positive and TUNEL-positive cells.

#### Percentage area occupied by GFAP-positive cells

In each of the groups (three sections per animal; 40 μm apart) the proportion of brain regions occupied by GFAP-positive astrocytes was assessed (objective: 40×; field size 0.04 mm^2^) throughout the striatum (five random fields of view), motor-somatosensory cortex (five fields of view), corpus callosum, cingulum (two fields of view) and external capsule (three fields of view). The proportion of staining in each section was totalled and averaged, and was expressed as a percentage of the brain region.

### Real-time polymerase chain reaction for human ALU sequences

Brains from LPS + hyperoxia pups (*n* = 2) and LPS + hyperoxia + hAEC pups (*n* = 3) were collected as already detailed. Then 10 mg of brain tissue were weighed out and RNA extracted using the PureLink Genomic DNA Mini Kit (Invitrogen). Real-time polymerase chain reaction (PCR) was performed with the use of the Applied Biosystems Power SYBR Green PCR Master Mix and the Rotorgene RG-3000 (Corbett Research, Australia). A 100 ng sample of total DNA was used in the PCR assay under the following cycling conditions: 95 °C for 10 min, then 35 cycles of 95 °C for 15 sec, 68 °C for 30 sec and 72 °C for 30 sec. The ALU primer sequence used was forward 5′-CATGGTGAAACCCCGTCTCTA-3′ and reverse 5′-GCCTCAGCCTCCCGAGTAG-3′. The PCR products were resolved by electrophoresis in 2% agarose gels, and the bands were visualized under UV light. DNA from 1 × 10^6^ hAECs were used as a positive control (+ve).

### hAEC cell tracking by immunofluorescence

Cell tracking of hAECs was performed using anti-human HLA-G (HLA-G clone 87G, 1:100, ALX-805-719-c100; Enzo Lifesciences) on 10-μm-thick brain sections from LPS + hyperoxia treated (*n* = 3) and untreated (*n* = 3) animals. Secondary antibody (1:200) was applied for 1 h at room temperature in a humid chamber.

### Primary microglia isolation using magnetic isolation

Primary microglia cell cultures were isolated using the EasySep® Mouse CD11b Positive Selection Kit (STEMCELL Technologies, Vancouver, Canada) as per the manufacturer's instructions. Briefly, brains were isolated from healthy, untreated CX3CR1^GFP/+^ P14 mice (minimum *n* = 3 per experimental group) and the meningeal layer removed and washed in ice-cold Dulbecco's modified Eagle medium (DMEM) supplemented with high glucose (DMEM/F12-High Glucose; Life Technologies, Australia) and 1% penicillin–streptomycin (PS; Life Technologies, Australia). Brains were then mechanically dissociated using fine scissors and triturated using a pipette. The subsequent single-cell suspension was passed through a 40-μm cell strainer. Cells were then pelleted and suspended in 1 ml of DMEM-F12 to obtain a final concentration of no more than 1.0 × 10^8^ cells/ml. The cell suspension was then transferred to a Falcon™ 5-ml polystyrene round-bottom tube (BD Biosciences, San Jose, CA, USA). Then 50 μl of rat serum was added to the sample, followed by incubation with EasySep® Selection Cocktail for 5 min. The tube was then placed into an EasyEights™ magnet for 10 min, and the supernatant carefully collected by pipette and discarded. This magnetic separation process was repeated three times (1 × 10 min, 2 × 5 min) to enhance the purity of the collected cells, and the sample allowed to recover overnight before proceeding with in-vitro studies. Culture purity was verified by flow cytometry from a subset of animals used (*n* = 5) with the microglia purity (CD45^low^Cd11b^+^) determined as >88%. Experimental groups consisted of microglia cultured in control media alone (DMEM/F12-High Glucose with L-glutamine, FBS and antibiotics), hAEC-conditioned media, LPS and LPS with subsequent culture in hAEC-conditioned media. Microglia were exposed to an inflammatory stimulus by culturing in media containing 1 μg/ml LPS (L2630; Sigma-Aldrich) for 48 h prior to experimental treatments.

### Collection of hAEC-conditioned media

Freshly thawed hAECs (0.5 × 10^6^) were seeded into a T175 flask (353028; BD Falcon®, Bedford MA, USA) with 20 ml DMEM/F12 (11320033; Invitrogen) supplemented with 10% heat-inactivated FBS (16000-036; Invitrogen) and penicillin–streptomycin (15070-063; Invitrogen). hAECs were cultured at 37 °C in 5% CO_2_ for 4 days. The conditioned medium was then collected and filtered through a 0.2-μm pore filter (16532; Sartorius Stedim Biotech, Goettingen, Germany) prior to use.

### Flow cytometric analysis

#### Staining protocol for M1/M2 phenotyping

Primary microglia (*n* = 3 animals for each group) were incubated with an Fc-receptor blocker (553141; BD Pharmingen) for 15 min on ice and stained with the following antibodies: CD45-V450 (1:100, 560541; BD Biosciences, North Ryde, NSW, Australia), Cd11b-PE (1:100, 12-0112; eBioscience, San Diego, CA, USA), CD86-phycoerythrin cyanine 7 (PE Cy-7, 1:200, 560501; BD Bioscience) and CD206-Alexa Fluor 647 (1:200, 12310; Australian Biosearch, Karrinyup, WA, Australia). All samples were run in duplicate, and flow cytometric data were collected on a BD FACS Canto II Analyzer (BD Biosciences) and analysed using FlowJo cytometric analysis software (Tree Star, Ashland, OR, USA).

### MTS assay

Microglia viability and proliferation was assessed using the Promega MTS assay (G5421; Promega), according to the manufacturer’s instructions. Briefly, 5 × 10^3^ microglia were plated into each well of a 96-well plate (BD Falcon). Primary microglia were stimulated with LPS with or without hAEC-conditioned medium (*n* = 3 animals for each group). For each treatment and time-point, 20 μl of MTS solution was added, and microglia were incubated for 4 h. Optical density was measured at 490 nm with the SpectraMax i3 Spectrophotometer (Molecular Devices, CA, USA). All samples were run in triplicate and analysed using the built-in SoftMax Pro software suite.

#### Phagocytic assay

The phagocytic activity of microglia was determined using a pHrodo assay as described previously [24]. Microglia were plated in 24-well flat-bottom culture plates (BD Falcon, Australia) at a density of 1.5 × 10^5^ cells per well overnight at 37 °C and 5% CO_2_. Microglia were then stimulated with or without LPS and hAEC-conditioned medium (*n* = 3 animals for each group). pHrodo® Red *E. coli* BioParticles® (10 μg/ml, 10025; Life Technologies, Australia) were added to each well and phagocytic activity analysed by fluorescence-activated cell sorting (FACS) after 30-min incubation at 37 °C. Negative controls were pre-chilled in ice to inhibit membrane movement. All samples were run in duplicate, and flow cytometric data were collected on a BD FACS Canto II Analyzer (BD Biosciences) and analysed using FlowJo cytometric analysis software (Tree Star).

### Apoptosis assay

Primary microglia were plated at a density of 1 × 10^5^ cells per well in a 24-well plate (BD Falcon) and stimulated with LPS with or without hAEC-conditioned medium (*n* = 5 animals for each group). Microglia were removed by aspiration and stained using the Annexin V:PE Apoptosis Detection Kit (BD Biosciences) according to the manufacturer’ s instructions. Propidium iodide (PI) was used to stain dead cells. Early-stage and late-stage apoptotic microglia were identified by use of the BD FACS Canto II Analyzer (BD Biosciences) and analysed using FlowJo 8.7 software (Tree Star).

### Cytokine assays

hAECs were thawed and seeded in T175 flasks overnight at 37 °C and 5% CO_2_. The following morning, cells were lifted using 0.05% trypsin and seeded at a density of 0.5 × 10^6^ cells/well in a 24-well plate (BD Falcon). Cells were stimulated with LPS (100 ng/ml) for 24 h and supernatant collected and spun down briefly to remove particulates (500 × *g* for 5 min). Levels of IL-6 (SKU0029; ELISAkit, Melbourne, VIC, Australia), IL-10 (SKU0007; ELISAkit), IL-37 (ELH-1L1F7; RayBioTech, GA, USA) and IL-1Ra (ab100565; abcam, Cambridge, UK) were measured in duplicate following the manufacturers’ instructions and quantified on a SpectraMax i3 Spectrophotometer (Molecular Devices).

### Statistics

Data were expressed as mean ± standard error of the mean (SEM). Differences between groups were analysed by an unpaired one-way analysis of variance unless otherwise stated. For differences assessed by one-way ANOVA, Newman–Keuls post-hoc correction was applied where appropriate to correct for multiple comparisons. All analysis was done using GraphPad Prism (GraphPad Software Inc, CA, USA). Data were considered significant when *p* < 0.05.

## Results

There were no significant differences between saline alone animals and saline + hAEC-treated pups raised in normoxia for any of the physiological or immunohistochemical outcomes. Therefore, only outcomes relating to LPS + hyperoxia pups with (*n* = 7) or without hAECs (*n* = 6) are compared with saline alone (control) animals (*n* = 9).

### Body and brain weights

**Fig. 1:**
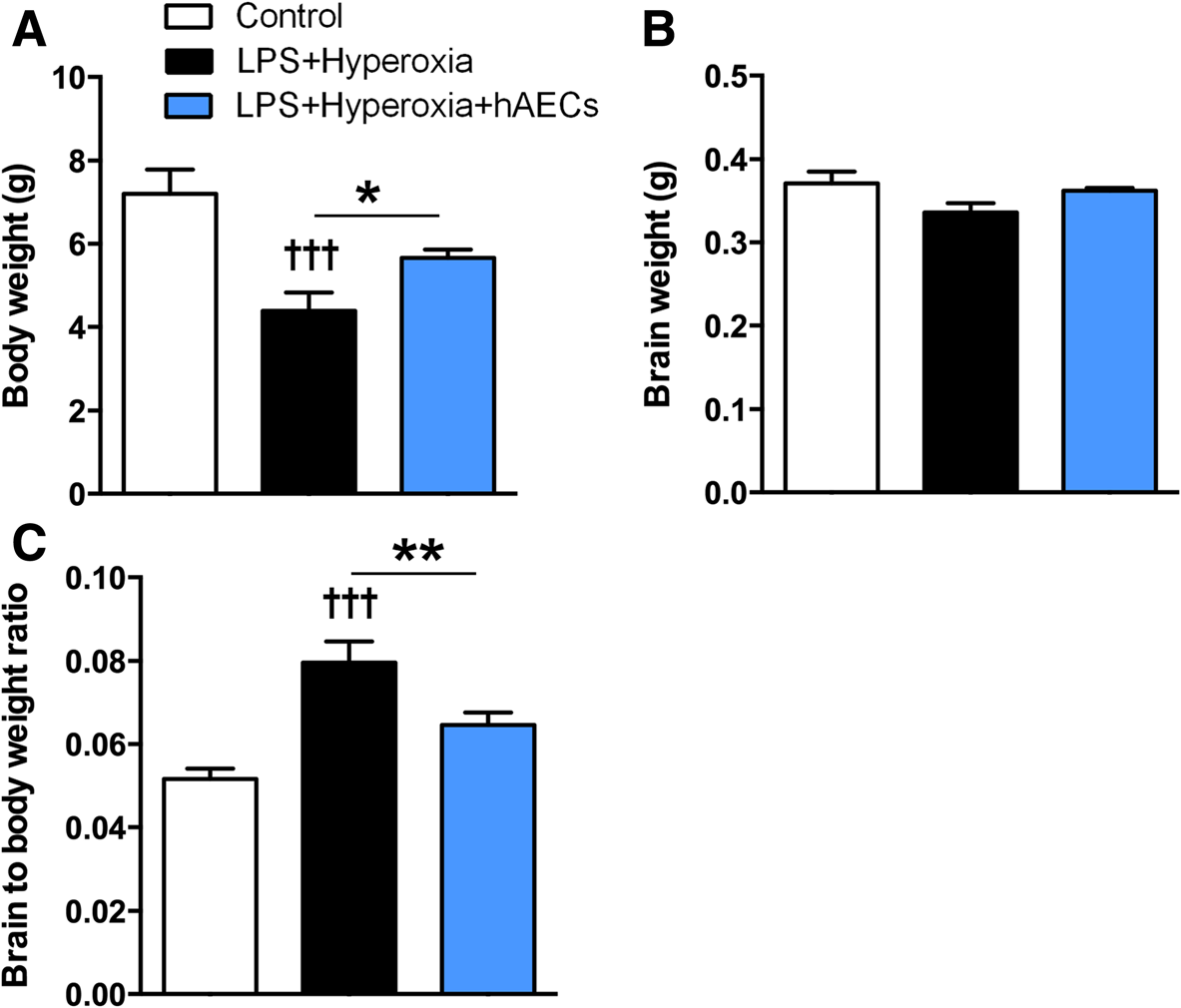
Brain and body weights in LPS and hyperoxia-treated animals, and effects of hAEC administration. **a** Body weights were significantly reduced by LPS + hyperoxia, with hAEC treatment reversing this change. **b** Brain weights were unchanged in the hyperoxia groups. **c** Brain to body weight ratio was increased in LPS + hyperoxia animals compared with controls, but was restored to control levels after hAEC treatment. *p < 0.05; **p < 0.01; †††LPS + hyperoxia versus control, *p* < 0.001. *hAEC* human amnion epithelial cell, *LPS* lipopolysaccharide

At P14 the mean body weight of LPS + hyperoxia animals was significantly reduced compared with controls (Fig. 1a, *p* < 0.001; controls, *n* = 9; LPS + hyperoxia, *n* = 6; LPS + hyperoxia + hAECs, *n* = 7), an effect normalized by hAEC administration (*p* < 0.05 vs LPS + hyperoxia, *p* > 0.05 vs control). There was no difference in brain weight between groups (Fig. 1b). However, the brain to body weight ratio was significantly increased in LPS + hyperoxia animals compared with both controls and LPS + hyperoxia + hAEC animals (Fig. 1c, *p* < 0.001 and *p* < 0.01, respectively).

### Gross structural analysis of the brain

Qualitative assessment of key brain structures in haematoxylin and eosin-stained slides revealed no evidence of gross morphological abnormalities (e.g. lesions, infarcts, necrosis, hydrocephalus or areas of pallor) in any experimental groups.

### hAECs in the brain

**Fig. 2:**
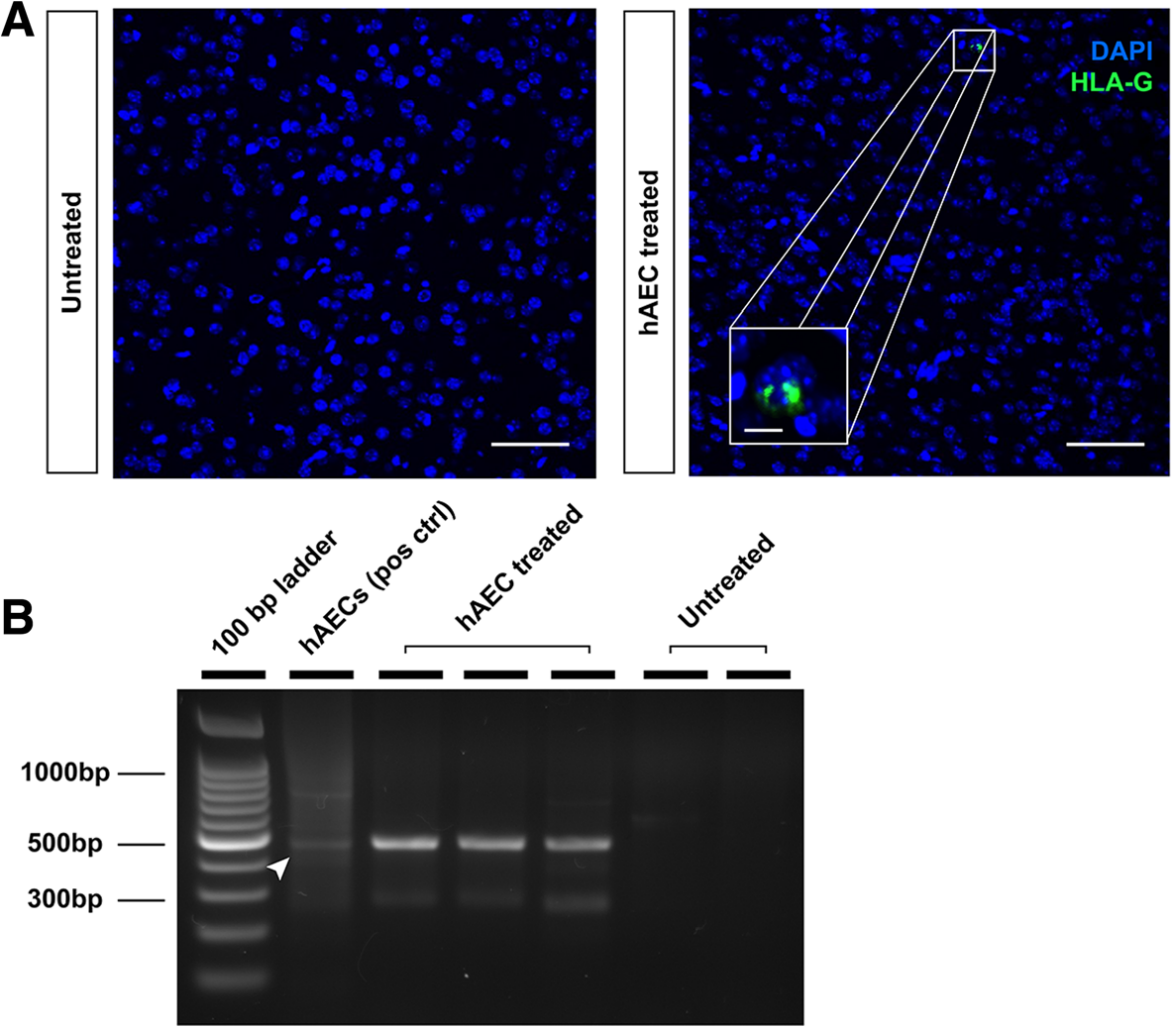
Tracking of hAECs within the brain. **a** Representative staining of DAPI (nuclear stain, *blue*) and HLA-G (*green*). HLA-G immunoreactive fragments were detected in cerebral cortex of 14-day-old hAEC-treated animals. *Scale bar* = 50 μm for main image, 3 μm for inset. **b** Gel electrophoresis of PCR products amplified from DNA extracted from the brains of treated and untreated LPS + hyperoxia animals. From left to right: Promega 100 bp DNA ladder, hAECs (positive control), hAEC-treated animals, untreated animals (Colour figure online). *hAEC* human amnion epithelial cell

HLA-G immunoreactivity was observed in the cerebral cortex, white matter and striatum (Fig. 2a). We also detected the presence of human DNA within the brains, as shown by amplification of human ALU repeats by real-time PCR (Fig. 2b; LPS + hyperoxia + hAEC animals, *n* = 3; LPS + hyperoxia alone, *n* = 2).

### Apoptosis

**Fig. 3:**
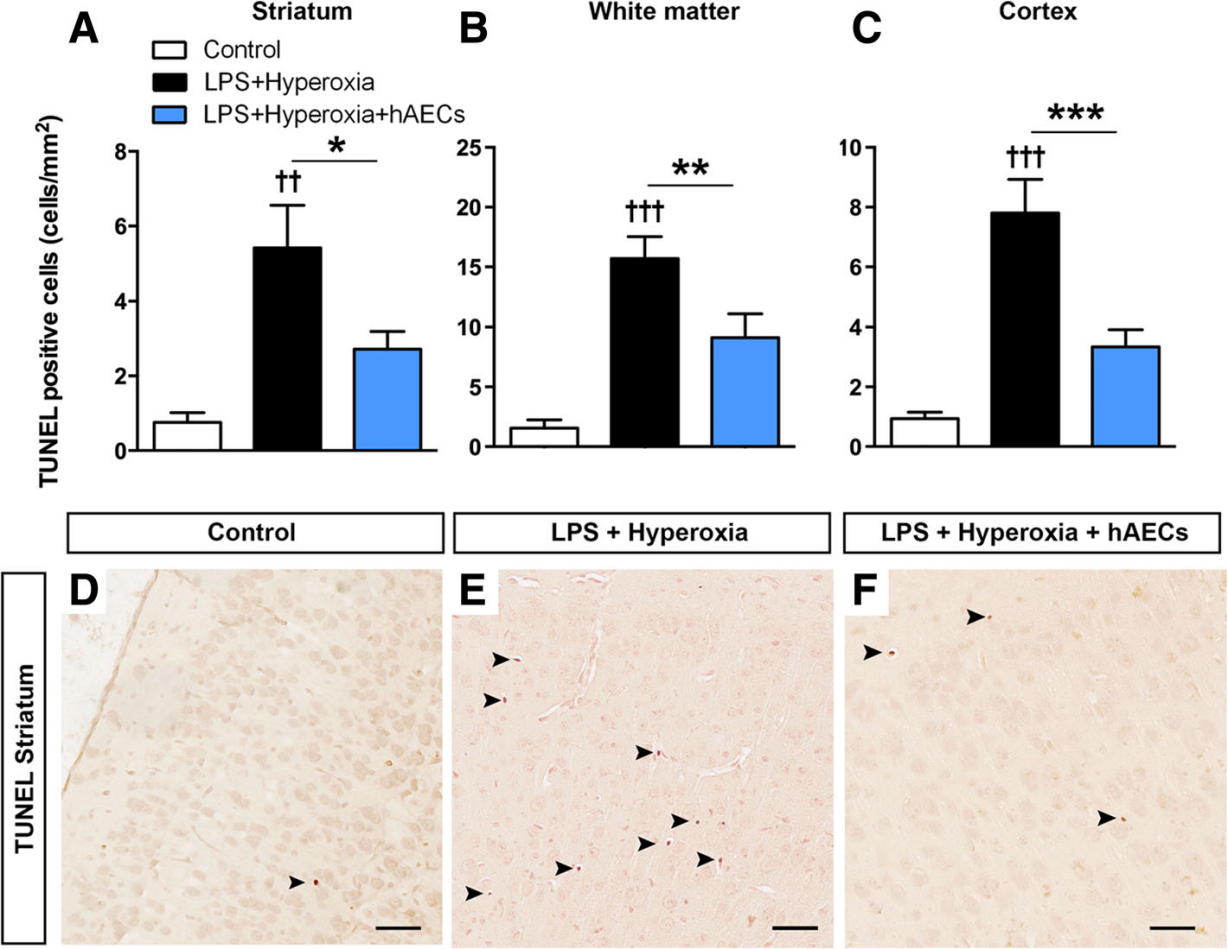
Density of terminal deoxynucleotidyl transferase 2′-deoxyuridine, 5′-triphosphate nick end labelling (*TUNEL*) apoptotic cells in the brain. Density of TUNEL cells was increased in the striatum (**a**), white matter tracts (**b**) and cerebral cortex (**c**) of LPS + hyperoxia animals. Representative figures showing no TUNEL-positive cells in a control animal (**d**) and increased TUNEL-positive cells (as indicated by black arrows) exhibiting nuclear condensation in an LPS + hyperoxia animal (**e**), which was reduced by hAECs (**f**). *p < 0.05; **p < 0.01; ***p < 0.001; ††LPS + hyperoxia versus control, *p* < 0.01; †††LPS + hyperoxia versus control, *p* < 0.001. *Scale bar* (**d–f**) = 30 μm. *hAEC* human amnion epithelial cell, *LPS* lipopolysaccharide

We performed the TUNEL assay to identify cells in late apoptotic stages exhibiting extensive DNA degradation in the striatum, white matter and cortex, because these are regions known to be susceptible to damage in preterm brain injury [39]. Cells that were TUNEL-positive were stained brown by the diaminobenzidine (DAB) chromogen and the majority of these cells displayed nuclear condensation, a well-identified morphological characteristic of cells undergoing apoptosis [40]. There was an increase in the density of TUNEL-positive cells in the striatum (Fig. 3a, *p* < 0.01; controls, *n* = 9; LPS + hyperoxia, *n* = 6; LPS + hyperoxia + hAECs, *n* = 7), subcortical white matter (Fig. 3b, *p* < 0.001) and motor-somatosensory cortex (Fig. 3c, *p* < 0.001) in LPS + hyperoxia animals compared with controls. Administration of hAECs to LPS + hyperoxia animals reduced TUNEL-positive cell density back to near control levels in all brain regions in LPS + hyperoxia animals (Fig. 3a–c; striatum, *p* < 0.05; white matter, *p* < 0.01; cortex, *p* < 0.001), as illustrated by representative images taken from the striatum of control (Fig. 3d), LPS + hyperoxia (Fig. 3e) and LPS + hyperoxia + hAEC (Fig. 3f) animals.

### Astrocytes

**Fig. 4:**
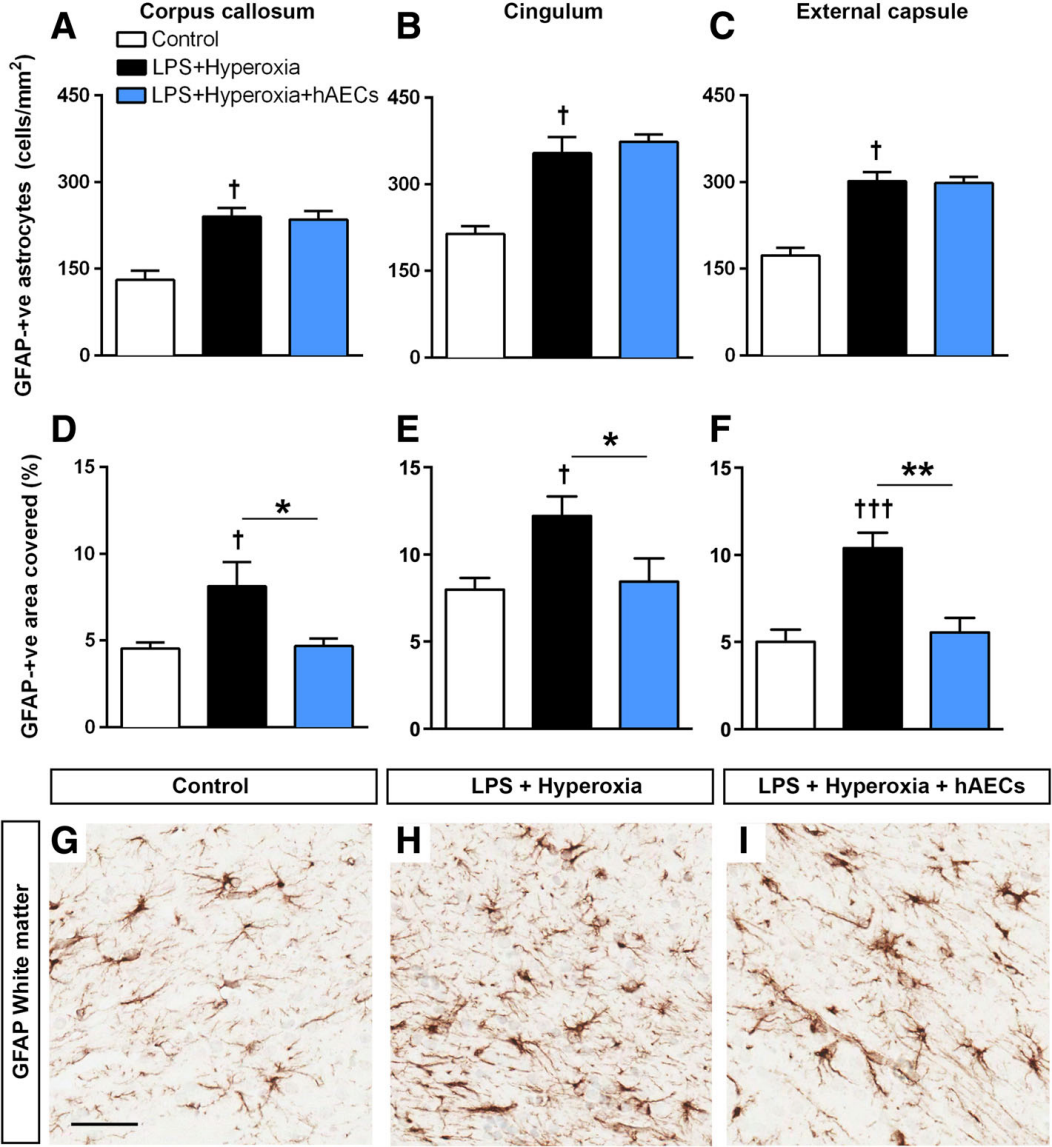
Assessment of astrocytosis in the cerebral white matter tracts. Density of GFAP-IR astrocytes in the corpus callosum (**a**), cingulum (**b**) and external capsule (**c**) was increased in LPS + hyperoxia animals compared with controls. hAEC treatment did not alter the density of GFAP-IR astrocytes in all regions (**a**–**c**). Percentage area covered by GFAP staining was increased in the corpus callosum (**d**), cingulum (**e**) and external capsule (**f**) in LPS + hyperoxia animals compared with controls. Interestingly, hAEC treatment reduced the percentage area covered by GFAP staining in all regions (**d**–**f**). Representative figures showing GFAP staining in the external capsule of control (**g**), LPS + hyperoxia (**h**) and LPS + hyperoxia + hAEC (**i**) animals. *p < 0.05; **p < 0.01; †LPS + hyperoxia versus control, *p* < 0.05; †††LPS + hyperoxia versus control, *p* < 0.001. *Scale bar* (**g**–**i**) = 150 μm. *GFAP* glial fibrillary acidic protein, *hAEC* human amnion epithelial cell, *LPS* lipopolysaccharide

The density of GFAP-positive cells in the corpus callosum (Fig. 4a), cingulum (Fig. 4b) and external capsule (Fig. 4c) was increased in LPS + hyperoxia pups compared with controls (*p* < 0.05; controls, *n* = 9; LPS + hyperoxia, *n* = 6; LPS + hyperoxia + hAECs, *n* = 7), an effect not mitigated by hAEC administration. We then assessed the proportion of the brain region occupied by GFAP immunostaining to take into account an effect on astroglial hypertrophy. There was an increase in the area coverage of GFAP immunoreactivity in the corpus callosum (Fig. 4d, *p* < 0.05), cingulum (Fig. 4e, *p* < 0.05) and external capsule (Fig. 4f, *p* < 0.001) in LPS + hyperoxia animals compared with controls. Treatment with hAECs mitigated this increase in all regions (corpus callosum and cingulum, *p* < 0.05; external capsule, *p* < 0.01; Fig. 4d–f), as shown by representative images of the external capsule of control (Fig. 4g), LPS + hyperoxia (Fig. 4h) and LPS + hyperoxia + hAEC (Fig. 4i) animals.

### Microglia/macrophages

**Fig. 5:**
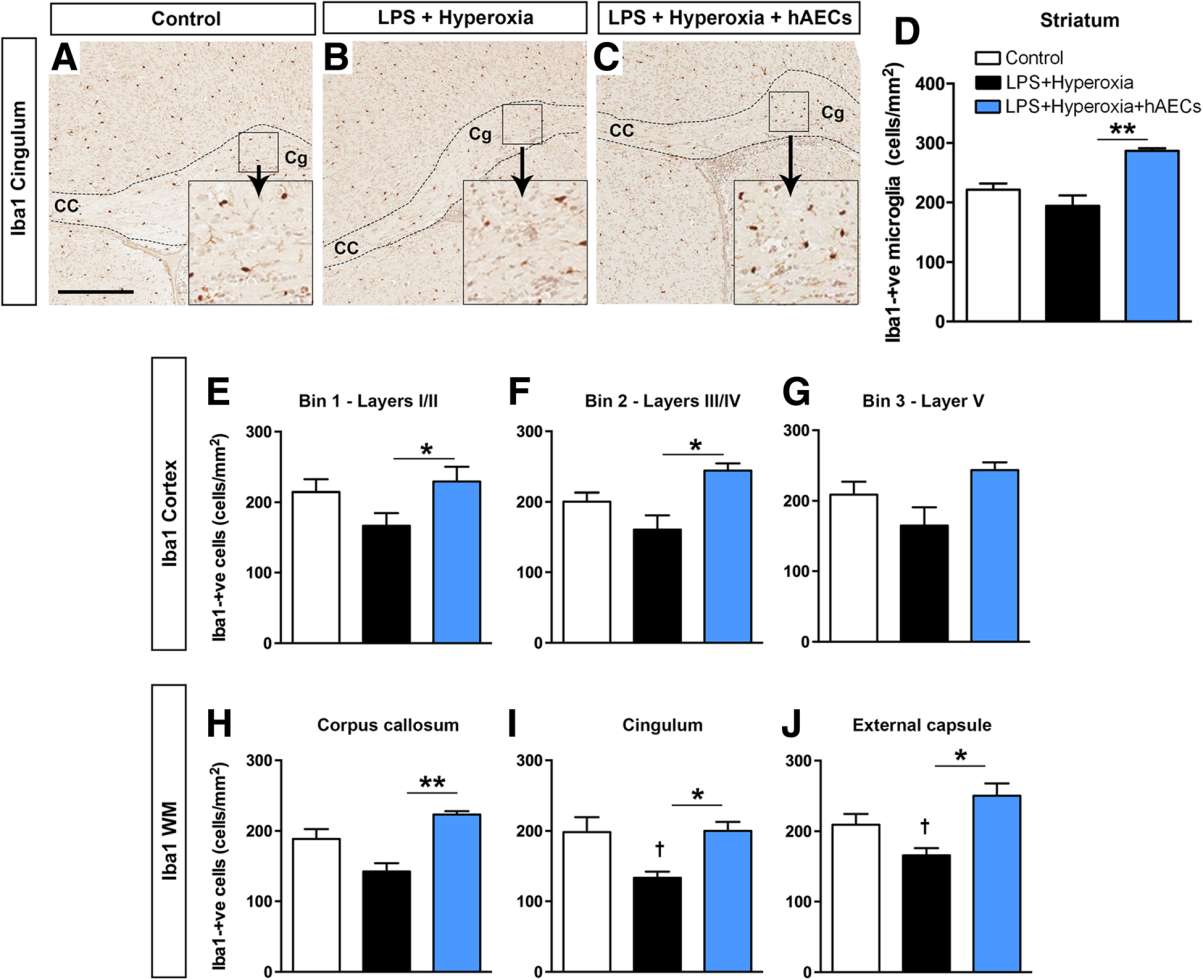
Assessment of the microglial response in the striatum. Representative images illustrating Iba1 immunoreactivity in the cingulum of control (**a**), LPS + hyperoxia (**b**) and LPS + hyperoxia + hAEC (**c**) animals. **d** Density of Iba1-positive cells in the striatum was not different between control and LPS + hyperoxia animals but was increased with administration of hAECs. **e**–**g** Assessment of the microglial response in the cerebral cortex. Density of Iba1-positive cells was not different between control and LPS + hyperoxia animals in all three bins, but was increased with administration of hAECs in bin 1 (**e**) and bin 2 (**f**). **h**–**j** Assessment of the microglial response in the white matter tracts. Density of Iba1-positive cells was not different between control and LPS + hyperoxia animals in the corpus callosum (**h**) but was reduced in the cingulum (**i**) and external capsule (**j**).*p < 0.05; **p < 0.01; †LPS + hyperoxia versus control, *p* < 0.05. *Scale bar* (**a–c**) = 200 μm. *CC* corpus callosum, *Cg* cingulum, *hAEC* human amnion epithelial cell, *LPS* lipopolysaccharide

We assessed the density of microglia/macrophages in the striatum, cortex and white matter, as shown by the representative figures (Fig. 5a–c). In the striatum, there was no difference in the areal density of Iba1-positive cells in LPS + hyperoxia animals compared with controls. Administration of hAECs significantly increased Iba1-positive cell density (Fig. 5d, *p* < 0.01; controls, *n* = 9; LPS + hyperoxia, *n* = 6; LPS + hyperoxia + hAECs, *n* = 7).

In the motor-somatosensory cortex, Iba1-positive cell density was not affected by LPS and hyperoxia (Fig. 5e-g). As in the striatum, administration of hAECs increased Iba1-positive cell density in bins 1 and 2, corresponding to cortical layers I/II and layers III/IV (Fig. 5e, f, *p* < 0.05).

The density of Iba1-positive cells in the corpus callosum in LPS + hyperoxia animals was not different from that in controls (Fig. 5h, *p* > 0.05), but was significantly decreased in the cingulum (Fig. 5i, *p* < 0.05) and the external capsule (Fig. 5j, *p* < 0.05). As for the striatum and cortex, hAEC administration to LPS + hyperoxia pups significantly increased the density of Iba1-positive cells in the corpus callosum (*p* < 0.001), cingulum (*p* < 0.05) and external capsule (*p* < 0.05).

**Fig. 6:**
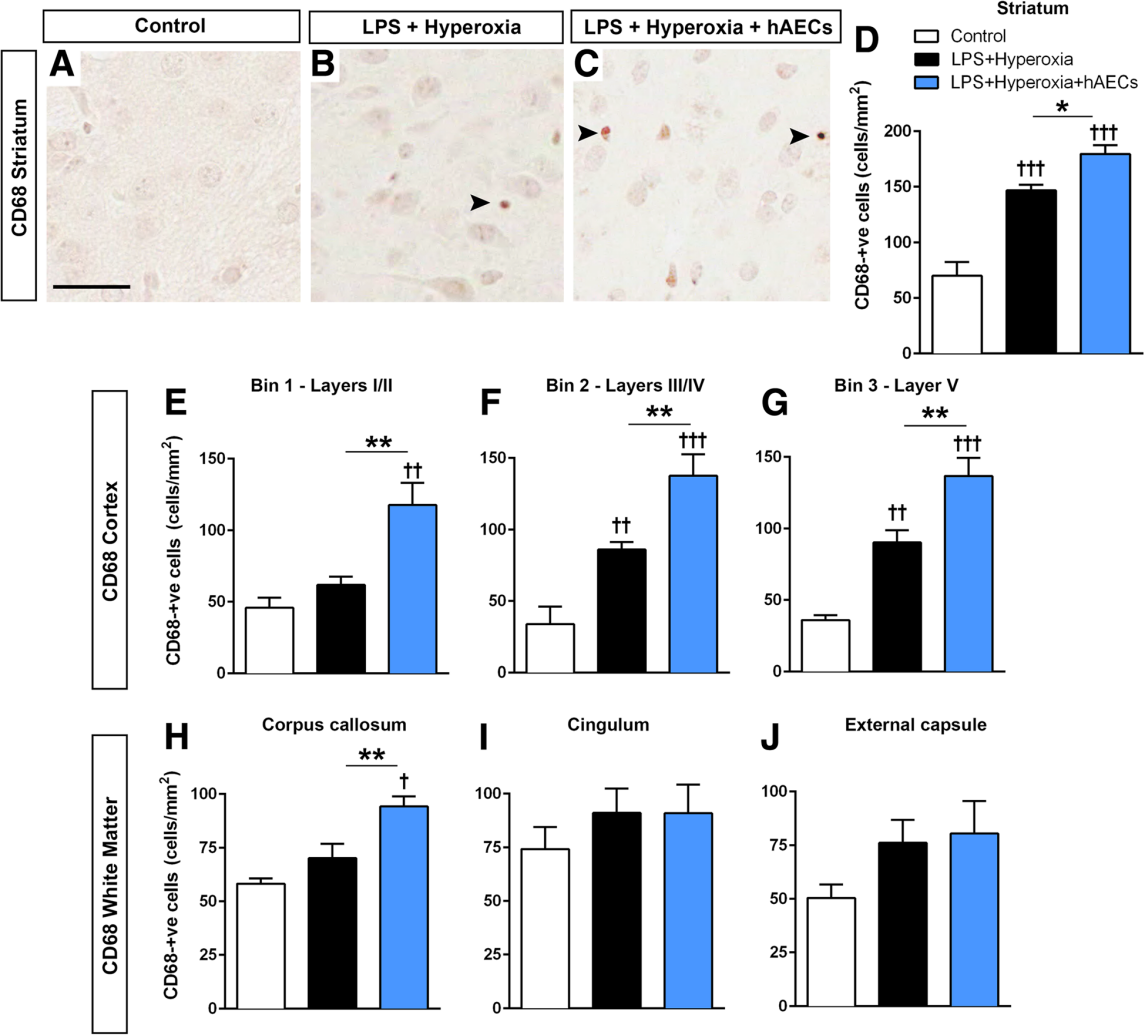
Assessment of microglial activation in the striatum. Representative images showing CD68^+^ activated microglia/macrophages in the striatum of control (**a**), LPS + hyperoxia (**b**) and LPS + hyperoxia + hAEC (**c**) animals. LPS and hyperoxia significantly increased the density of CD68^+^ activated microglia/macrophages, and this was further increased in hAEC-treated animals (**d**). **e**–**g** Assessment of microglia/macrophage activation in the cerebral cortex. Density of CD68^+^ cells was not different between control and LPS + hyperoxia animals in bin 1 (**e**), but was increased in bin 2 (**f**) and bin 3 (**g**). Administration of hAECs to LPS + hyperoxia animals increased the density of CD68^+^ cells activated in all three bins (**e**–**g**). **h**–**j** Assessment of CD68 staining in the cerebral white matter tracts. Density of CD68^+^ cells was not different between control and LPS + hyperoxia animals in the corpus callosum (**h**), cingulum (**i**) or external capsule (**j**); administration of hAECs increased the density of CD68^+^ cells in the corpus callosum (**h**). *p < 0.05; **p < 0.01; ††LPS + hyperoxia versus control, *p* < 0.01; †††LPS + hyperoxia versus control, *p* < 0.001. *Scale bar* (**a–c**) = 25 μm. *Black arrows*, CD68^+^ cells.*hAEC* human amnion epithelial cell, *LPS* lipopolysaccharide

We next quantified the density of activated microglia/macrophages using the marker CD68 as shown by representative images (Fig. 6a-c), because Iba1 labels both resting and activated states [41, 42]. In the striatum, the density of CD68-positive activated microglia/macrophages was significantly increased in LPS + hyperoxia animals compared with controls (Fig. 6d, *p* < 0.001; controls, *n* = 9; LPS + hyperoxia, *n* = 6; LPS + hyperoxia + hAECs, *n* = 7) and further increased in LPS + hyperoxia + hAEC animals (Fig. 6d, *p* < 0.05). We observed a similar effect across the three groups of animals in the cerebral cortex (Fig. 6e, f), with an increase in the density of CD68-positive cells in LPS + hyperoxia pups compared with controls (bins 2 and 3 only; *p* < 0.01 for both) and a further increase in hAEC-treated animals across all three regions (*p* < 0.01 for all groups). We observed no significant differences in CD68-positive cell density in the corpus callosum, cingulum and external capsule in LPS + hyperoxia animals compared with controls (*p* > 0.05). However, compared with both control and LPS + hyperoxia animals, LPS + hyperoxia + hAEC pups had a significant increase in CD68-positive cell density in the corpus callosum (Fig. 6h–j; *p* < 0.05 vs control, *p* < 0.01 vs LPS + hyperoxia).

### In-vitro assessment of microglial function after hAEC co-culture

**Fig. 7:**
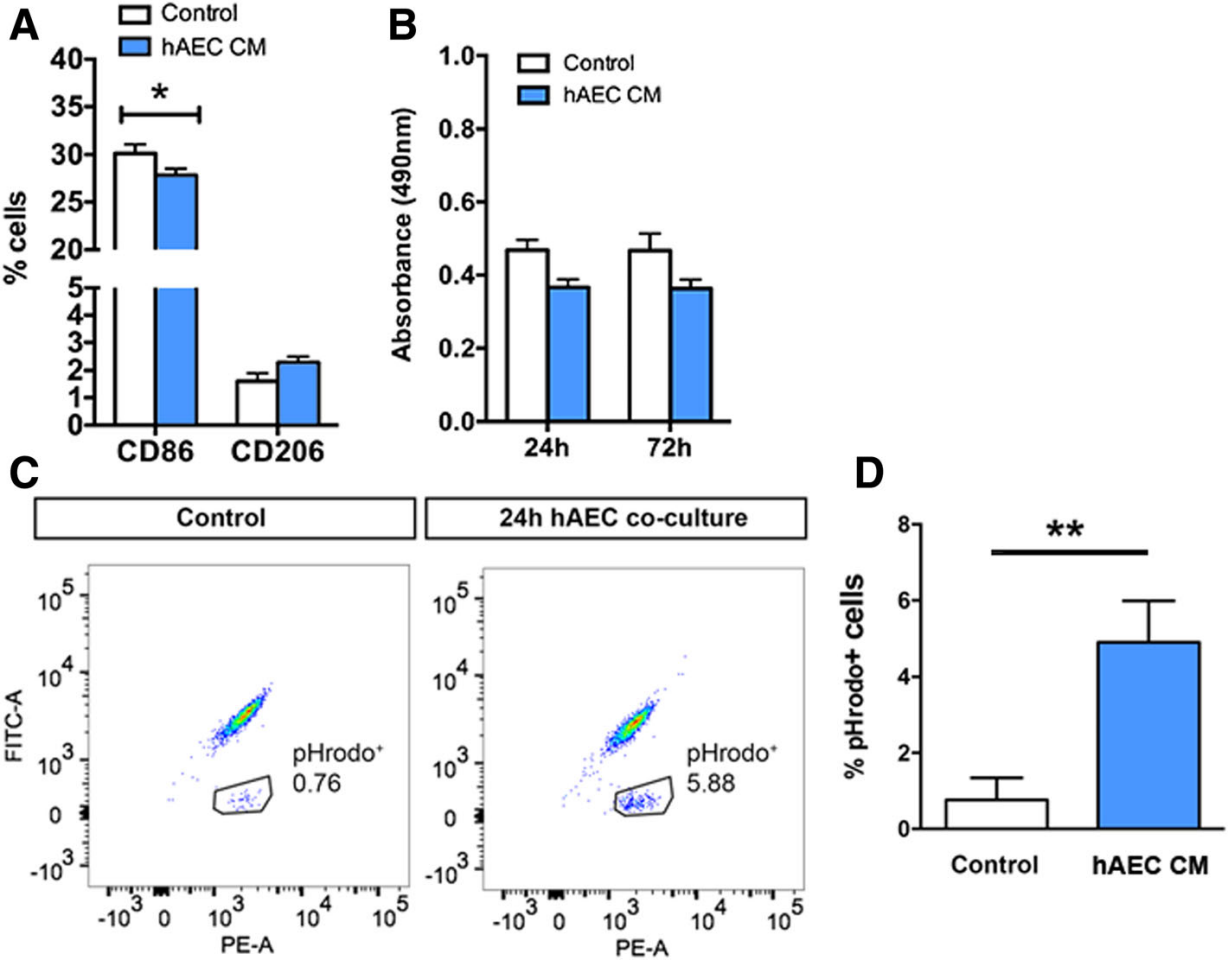
Effects of hAEC-conditioned medium treatment on primary microglia isolated from P14 animals, relative to controls treated with ultra-culture medium alone. **a** Percentage of microglia expressing the M1 marker CD86 and M2 marker CD206; 24-h exposure to hAEC-conditioned medium significantly reduced the percentage of CD86 expressing microglia. **b** Soluble factors from hAECs do not affect microglia proliferation after LPS stimulation at 24, 48 or 72 h. **c** Representative FACS plots illustrating the marked increase in pHrodo^+^ cells after 24-h hAEC co-culture. **d** After a 24-h co-culture of primary microglia with hAECs, the number of pHrodo^+^ cells increased significantly. *p < 0.05, **p < 0.01, Student’s *t* test. *hAEC* human amnion epithelial cell, *CM* conditioned medium

In light of our observations regarding increased density of total and activated microglia/macrophages following hAEC administration, we explored whether hAEC-conditioned medium could alter microglial function in vitro. To do this, we ran a series of in-vitro assays using primary microglia isolated from brains of healthy 14-day-old CX3CR1^GFP/+^ mice, which express GFP in all mononuclear phagocytes, and whose expression in the brain is restricted to microglia [43]. We then co-cultured these microglia with hAEC-conditioned medium, compared with controls treated with ultra-culture medium alone. Following 24 h of stimulation with LPS, co-culture of primary microglia with hAEC-conditioned medium significantly decreased the amount of CD86-positive M1-subtype microglia, as determined by FACS analysis (Fig. 7a; 30.1 ± 0.95% vs 27.9 ± 0.66% total cells, *n* = 3 each group, *p* = 0.0173, Student’s *t* test). hAEC-conditioned media had no effect on microglia proliferation, as assessed by the MTS assay, at 24, 48 or 72 h (Fig. 7b; *n* = 3 each group), but increased microglia phagocytic activity (Fig. 7c, d; 0.76 ± 0.58% vs 5.88 ± 0.60% total cells, *n* = 4 each group, *p* = 0.0328, Student’s *t* test).

**Fig. 8:**
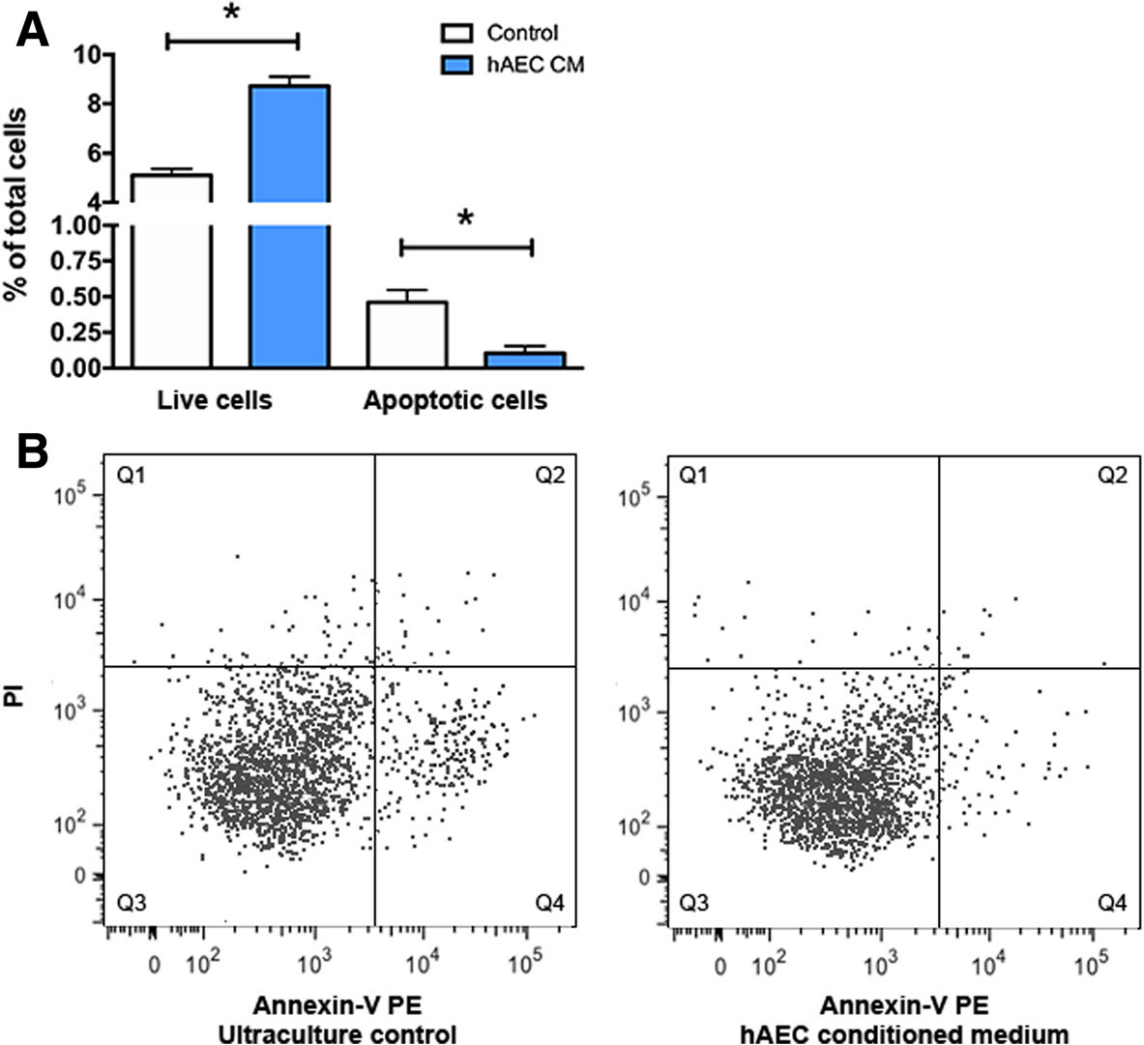
Soluble factors from hAEC protect microglia after LPS stimulation. **a** Following 24 h of LPS stimulation, 48-h exposure to hAEC-conditioned medium significantly increased the percentage of live microglia and reduced the percentage of apoptotic microglia. **b** Representative FACS plots demonstrating the shift of microglia into Q4 (Annexin-V^+^ PI^–^). *Q1* necrotic cells, *Q2* late apoptotic or dead cells, *Q3* live cells, *Q4* early apoptotic cells. *p < 0.05, Student’s *t* test. *hAEC* human amnion epithelial cell, *CM* conditioned medium

We next assessed microglia apoptosis after 24 h of LPS stimulation, with or without hAEC-conditioned medium for 24 h (Fig. 8). We found that hAEC-conditioned medium significantly increased the percentage of live microglia (CX3CR1^+^) compared with ultra-culture only controls (Fig. 8a; 5.1 ± 0.27% vs 8.7 ± 0.39% total cells, *n* = 5 each group, *p* = 0.0022, Student’s *t* test). This was accompanied with a decrease in percentage of apoptotic microglia (Fig. 8b; 0.46 ± 0.087% vs 0.10 ± 0.05% total cells, *n* = 5 each group, *p* = 0.0038, Student’s *t* test).

**Fig. 9:**
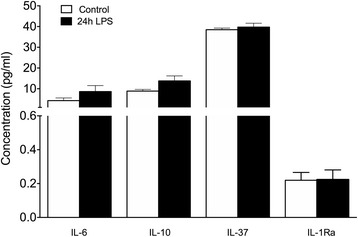
Release of cytokines from hAECs following 24-h LPS stimulation. Stimulation with LPS did not significantly alter the release of known microglia-modulating cytokines IL-6, IL-37, IL-10 or IL-1Ra. *LPS* lipopolysaccharide

Finally, we sought to determine whether the modulatory effect of hAECs on microglia was a result of the release of pro-inflammatory and anti-inflammatory cytokines known to have modulatory or polarizing roles on microglia and macrophages. Following 24-h stimulation with LPS, we found no significant differences in levels of IL-6, IL-10, IL-37 or IL-1Ra released by hAECs relative to unstimulated controls (Fig. 9; control, *n* = 10; LPS stimulation, *n* = 7; Student’s *t* test).

## Discussion

Using a mouse model of perinatal brain injury, induced by intrauterine inflammation and perinatal hyperoxia, we have shown that, when administered neonatally, hAECs are effective at reducing injury severity as evidenced by decreased apoptosis and astrogliosis. However, somewhat surprisingly, the administration of hAECs was associated with an increase in both microglial density and activation in the cerebral cortex, striatum and subcortical white matter. Exploring this further, we showed that co-culture of LPS-stimulated microglia with hAEC-conditioned medium reduced the percentage of CD86^+^ microglia, and increased microglial phagocytic activity. While hAEC-conditioned medium did not affect microglia proliferation, it did increase the percentage of live, LPS-challenged microglia after 48 h, with a corresponding decrease in percentage of apoptotic microglia compared with cells cultured in control medium alone. These observations suggest that hAECs may be an effective neuroprotective cell therapy acting via immunomodulation of microglia.

### A two-hit model to mimic neonatal brain injury

Infection and respiratory compromise are thought to be common and important antecedents of subsequent brain injury in extreme preterm infants, regardless of whether the injury arises before or shortly after birth [44, 45]. Previous work by others indicated an intermediate “tolerance” window about 24–48 h after LPS administration during which mice were actually protected from hypoxia–ischaemic brain injury [46, 47]. After this “tolerance” window, brains are primed from LPS pre-treatment and neural injury is increased instead. In a mouse model of HI and combined LPS, the degree of brain injury was more severe with LPS pre-treatment 72 h before HI than with just HI alone [48]. In our mouse model we sought to mimic this “two-hit” injury and thus administered LPS intra-amniotically at E16, 5 days before exposure to hyperoxia and thus outside this tolerance window, confirming that the combined insult induces subtle brain injury. The effects of hyperoxia in perinatal brain injury have not been researched extensively, with most studies applying hypoxia instead [3, 49]. However there is growing recognition that ventilation strategies which do not account for transient reduction in oxygen requirements, or prolonged continuous positive airway pressure, can increase oxidative stress in vital organs such as the lung and brains, and can result in the development of preterm infant complications such as perinatal brain injury [2]. Ventilation-induced injuries are further complicated by preterm infants already experiencing a several-fold increase in arterial oxygen concentration after birth, which could exacerbate injury. In fact, avoiding unnecessarily high oxygen saturation targets has been associated previously with improved outcomes specifically in preterm infants [50]. Given that hyperoxia has been shown to result in delayed white matter development and long-term white matter deficiency in a rat model [51], there is an urgent need to better understand the impact of hyperoxia and how we can prevent the development of long-term complications.

Here we report that LPS + hyperoxia exposure caused apoptosis in the striatum, cerebral white matter and motor-somatosensory cortex at P14. This supports previous reports using similar models [12, 26, 27] showing apoptotic injury with perinatal inflammation and hyperoxia-induced brain injury in mice. Astrogliosis was also evident 14 days after onset of perinatal hyperoxia, and nearly 20 days after the initial prenatal inflammatory insult. Astrogliosis occurs following brain injury [52] and is commonly associated with neural damage induced by cerebral hypoxia [53] and disruption of the fragile astrocyte–neuron relationship, resulting in reduction of dendritic arborization and increased synaptic loss [54, 55]. Increased astrocyte processes, or hypertrophy, as well as increased astrocyte cell numbers, or hyperplasia, are hallmarks of reactive astrogliosis seen in models of CNS injury or neurodegenerative disease, although there is clear evidence that astrocyte hypertrophy can occur independent of hyperplasia and is arguably more important in the pathology of CNS trauma [56]. Our observation of increased area coverage of GFAP reflects up-regulation of this intermediate filament protein in response to injury, and thus long-term transcriptional changes due to the continued stress [57]. This activates pathways leading to disturbed ion homeostasis, energy metabolism and glutamate release resulting in neuronal apoptosis. The persistence of astrogliosis could have implications for brain function, because astrocytes are both neurosupportive and neurodegenerative [58, 59]. This highlights the potential importance of proper management of assisted ventilation of preterm babies.

Microglial proliferation and/or activation are typical markers of inflammation and injury in the brain [7, 9, 60]. Studies utilizing similar animal models of either neonatal LPS administration [61] or chronic hyperoxia after birth [31, 62], albeit at much higher O_2_ concentrations (80% and 85% respectively), have reported increases in microglia density and activation. Here we report that at P14 there was an increase in the proportion of CD68-positive activated microglia/macrophages but no difference in the total density of Iba1-positive cells in animals that received prenatal LPS in combination with chronic perinatal hyperoxia. This heterogeneity in the microglial response to brain injury is interesting; however, it is likely that the differences across studies are attributable to both the length of time as well as window of onset of injury. In line with this reasoning, previous studies administering intraperitoneal LPS injections to pregnant rats at E13.5 reported no change in microglia density at 3 and 6 weeks of age respectively [63]. Another possibility is that we have missed the peak time-point for microglia expansion. Previous studies have reported that the peak microglial response to primary inflammatory-induced injury was approximately 5–14 days later [64], and between 1 and 2 weeks for secondary injury [63], which corroborates the lack of increase in microglia density at P14. The long-term functional implications of increased microglial activation in our model remain unknown, but other studies have shown that sustained microglial overactivation can induce demyelination [63] and neurodegeneration [65]. A recent study proposed that this neurotoxicity may be due to epigenetic changes in microglia after injury that persist into adulthood [66]. Taken together, these studies suggest that chronic inflammation at a critical window of development is sufficient to cause long-term impairment and thus swift intervention is required.

### Amnion cells as a therapy for brain injury

The reparative therapeutic potential of hAECs was highlighted by the recognition that they possessed stem cell-like characteristics [20, 36]. hAECs have been assessed in a multitude of other experimental animal models including bronchopulmonary dysplasia [17], cancer [67] and multiple sclerosis [68]. In a previous study using a hyperoxia-induced model of lung injury, hAECs significantly reduced tissue fibrosis and inflammatory markers when administered via intraperitoneal injection [17]. However, to date there has been only one study exploring their reparative effects in perinatal brain injury [19]. In that study, intrauterine LPS was used to mimic chorioamnionitis and subsequent brain injury in a fetal sheep model. The findings reported in the current study are broadly in line with the previous study in that hAEC administration in our mouse model of perinatal brain injury was also associated with less cell death and astrogliosis. Specifically, we observed reduced astroglial hypertrophy, as reflected by a reduction in GFAP area coverage, after hAEC administration. Reduction in astrocyte area coverage after injury typically marks a return to a non-reactive state, signalling a return to homeostasis [69]. However, in contrast to the previous report, we found that hAEC administration, intriguingly, increased microgliosis and activation in the striatum and white matter rather than decreasing it [19].

The reason for this apparent difference in microglial number and activation states between this study and previous findings is not clear. It is possible that it relates to the timing of cell administration relative to the insult. In the study by Yawno et al. [19], the hAECs were administered at the same time as LPS, almost in a preventative manner. Here, we administered hAECs after the combinatorial infection and hyperoxia had been allowed to establish for 4 days, which is a more likely scenario in clinic. Similar experiments using hAECs to prevent acute lung injury have also shown that administration immediately after the insult prevents macrophage migration into the lung [24, 70], whereas inflammation was not suppressed when hAECs were given 7 days after the insult [71]. Further assessment of the timing of hAEC administration and their effectiveness in preventing or repairing brain injury is certainly merited.

Here, we observed HLA-G immunoreactivity within the white matter, striatum and cortex of the hAEC-treated LPS + hyperoxia animals, 10 days after hAECs were administered. We also detected the presence of human ALU repeat sequences within the brain, suggesting the presence of human DNA. Whilst the HLA-G staining co-localized with DAPI, given the shape and size of the stain, it is likely that we are detecting amnion cell fragments that have entered the brain. This corroborates previous work using CFSE labelling showing that hAECs are able to cross the blood–brain barrier after being administered via intravenous injection in both a sheep model of brain injury [19] and a similar hyperoxia-induced mouse model of lung injury [17]. While we are unable to confirm whether hAECs enter the brain fully intact or after fragmentation, our work and other studies have demonstrated that the effects of hAECs are probably due to trophic factors released by hAECs rather than direct engraftment [24, 72]. Specifically, we demonstrated previously that hAEC-conditioned medium is sufficient to modulate the activity of lung macrophages, without the need for cell-to-cell contact with amnion cells [22]. In this model, we noted increased CD68 (or ED-1) expression after hAEC administration, which occurs during progressive activation of microglia away from their quiescent resting state, along with other markers such as vimentin [73, 74]. This is associated with increased phagocytosis as well as increased proliferation and expression of major histocompatibility complexes which are usually upregulated during disease resolution. Using in-vitro co-culture assays we thus sought to reproduce these findings in primary brain microglia. We observed significant modulation of microglia activation and survival, with hAEC-conditioned medium causing a reduction in CD86-positive microglia, increased phagocytosis and increased survival after LPS stimulation. The polarization and activation of microglia to either an M1 or M2 subtype in vivo is not biphasic, but rather a complex, constantly-evolving process [7]. Activation states will thus be highly dependent on the time-point at which the assay was performed. Given that the CD68 histological data were collected from brain tissue 10 days after hAEC administration and the M1/M2 polarization assay only 72 h after LPS stimulation, it is unsurprising that there was a disparity in the CD68 and CD86 findings (both are typically used as markers of the M1 subtype [75]). Nonetheless, we were able to conclude that hAEC-conditioned medium could affect microglia polarization in the acute phase.

The hAEC-induced changes in phagocyte polarization states have been reported previously. In a bleomycin-induced acute lung injury model we reported that hAECs can mediate macrophage polarization away from the pro-inflammatory M1 phenotype (CD86^+^) to the pro-reparative M2 phenotype (CD206^+^) [24]. It is well described in the literature that M2 microglia play critical roles in wound healing and repair [76], and are associated with reduced inflammation and increased phagocytic activity [77]. It is likely that the induction of phagocytosis is an important element of how hAECs facilitate injury repair, whether in the lung or brain. During injury, microglial activation and subsequent phagocytosis are widely regarded as critical for disease resolution [78] as a result of increased clearance of toxic debris and production of neurite growth-inhibiting compounds, both of which are vital for neuronal recircuitry and repair [79]. Further, activated microglia have been proposed to “phagoptose” live but stressed neurons that have been exposed to noxious stimuli, such as reactive oxygen species [80], to induce them to express “eat-me” signals, such as phosphatidylserine and calreticulin [81]. In our co-culture assays, we also observed increased microglia survival after inflammatory insult and subsequent treatment with hAEC-conditioned medium. Taken together, it is attractive to propose that hAECs are protecting microglia from inflammation-induced injury. This allows for sustained activation and phagocytosis of toxic apoptotic debris or phagoptosis of stressed-but-viable neurons, which in other neurodegenerative disorders has been shown to result in resolution of pathology.

Beyond these “clearance activities”, activated microglia are also thought to play a number of other roles in brain injury repair [82]. For example, they are able to drive oligodendrocyte differentiation during re-myelination [83], protect neurons during oxygen and glucose depletion in stroke models [84] and promote neurite outgrowth and arborization in a spinal cord injury model [85]. Our observations in this current study suggest that hAECs may be able to promote these functions via modulation of host microglia.

Cytokines are a key mediator of microglia function and activity [81]. As such, we assessed cytokine release from hAECs after LPS stimulation in vitro to elucidate potential glial modulatory pathways. We showed no differences in the levels of the LPS-inducible cytokine IL-6 [86] and the anti-inflammatory cytokines IL-10, IL-37 and IL-1Ra [87] after 24 h of LPS stimulation. IL-10 and IL-37 have been shown in the literature to polarize microglia towards the alternatively activated M2 subtype, which is pro-reparative [77, 88], as well as to induce microglia phagocytosis [89]. However, combined with our in-vivo data showing no effect of hAEC treatment for uninjured animals, the release of cytokines in an inflammatory environment is suggested not to be the major pathway by which hAECs modulate microglia. Indeed, previous studies suggest that hAECs mediate their reparative and neurotrophic properties via release of growth factors such as brain-derived neurotrophic factor and neurotrophin-3 [90]. Further research should attempt to elucidate the precise factors and pathways activated by hAECs in modulating microglia function and survival.

Cell therapies have been a particular focus for treating cerebral palsy in the past 5 years. Clinicaltrials.gov lists seven ongoing and completed trials that have utilized autologous stem cells derived from bone marrow or cord blood. Currently, our research group is conducting a first-in-human phase 1b safety trial for hAEC administration to infants with severe bronchopulmonary dysplasia (ACTRN12614000174684), and have been funded and granted approval to assess hAECs in adult liver cirrhosis (ACTRN12616000437460p). To progress the translation of hAECs into the clinic further, future studies should assess the long-term neuropathological and behavioural effects of hAECs in other preclinical models of perinatal brain injury. The aetiology of cerebral palsy is multifactorial, and thus showing therapeutic merit in multiple preclinical models of cerebral palsy encompassing different pathogenic pathways [49], as well as improvement in long-term outcomes, would greatly support the transition of hAECs to the clinic.

## Conclusions

We have generated a mouse model of perinatal brain injury combining prenatal LPS with chronic perinatal hyperoxia. We observed an increase in apoptosis in the striatum, cerebral white matter and cortex, as well as astrogliosis in the white matter tracts in 14-day-old animals. Administration of hAECs at P4 was effective at significantly reducing apoptosis and astrogliosis and was accompanied by microgliosis in the cortex, white matter and striatum. We also showed that in-vitro hAEC-conditioned medium can exert direct immunomodulatory effects on microglia. We demonstrate here that factors released from hAECs reduced M1 activation and increased phagocytic activity, consistent with the reduced apoptosis in LPS + hyperoxia + hAEC mice. Treatment with hAEC-conditioned medium also increased survival and reduced apoptosis of microglia after LPS stimulation, although it had no effect on microglia proliferation. While previous studies have assessed the therapeutic potential of hAECs in the brain, this is the first evidence that hAECs are able to modulate microglia within the brain, a property that does not appear to be mediated by release of cytokines by hAECs. These data suggest that hAECs have therapeutic utility in neonatal brain injury and the findings of this study add to the mounting evidence supporting their clinical use.

## Abbreviations

CNS: Central nervous system
E: Embryonic day
GFAP: Glial fibrillary acidic protein
GFP: Green fluorescent protein
hAEC: Human amnion epithelial cell
Iba1: Ionized calcium-binding adapter molecule 1
IR: Immunoreactive
LPS: Lipopolysaccharide
P: Postnatal day
TUNEL: Terminal transferase nick end-labelling
